# Clinical and Epidemiological Characteristics of an Oropouche Virus Outbreak in Loreto, Peru (October 2024–March 2025)

**DOI:** 10.3390/pathogens15010119

**Published:** 2026-01-21

**Authors:** Miguel Ángel Rojo-Pérez, Edgar A. Ramírez-García, Jara Llenas-García

**Affiliations:** 1 Internal Medicine Department, Hospital Universitario Cruces, 48903 San Vicente de Barakaldo, Spain; miguelangel.rojoperez@osakidetza.eus; 2 Department of Infectious and Tropical Diseases, Hospital Regional de Loreto “Felipe Santiago Arriola Iglesias”, Iquitos 16004, Peru; edgar.ramirez@unapiquitos.edu.pe; 3 Facultad de Medicina Humana, Universidad Nacional de la Amazonía Peruana (UNAP), Iquitos 16007, Peru; 4 Imported Diseases and International Health Unit, Hospital Universitario La Paz-Carlos III-IdiPaz, 28029 Madrid, Spain; 5 Biomedical Research Networking Center for Infectious Diseases (CIBERINFEC), Carlos III Health Institute, 28029 Madrid, Spain; 6 Clinical Medicine Department, Miguel Hernández University, 03202 Elche, Spain

**Keywords:** Oropouche virus, arbovirus, Peru, febrile illness, epidemiology, pregnancy, Oropouche fever

## Abstract

Oropouche virus (OROV) has emerged as a significant arboviral pathogen in South America, responsible for recurrent outbreaks of febrile illness. In the Loreto region of Peru, more than 600 cases were reported in 2024, markedly exceeding expected incidence rates. We conducted a retrospective observational study using clinical–epidemiological records of all RT-qPCR-confirmed cases of Oropouche fever from the Regional Health Directorate of Loreto between October 2024 and March 2025. A total of 100 confirmed cases were identified. The most frequent symptoms were fever (88%), headache (78%), and myalgia (72%). No atypical or neurological presentations were reported. No severe cases or deaths occurred. Eight patients required hospitalization, mainly due to severe abdominal pain, persistent vomiting, arthralgia, and pregnancy. Six pregnant women were identified; three experienced pregnancy complications, though no fetal malformations or miscarriages were observed. This outbreak represents a new OROV epidemic in the region, with fewer cases than in 2024 and predominantly mild clinical courses. Although outcomes were generally favorable, the occurrence of complications in pregnant women underscores the importance of continued molecular surveillance and targeted public health interventions.

## 1. Introduction

Oropouche virus (OROV) is an emerging orthobunyavirus responsible for recurrent outbreaks of febrile illness in tropical regions of Latin America. First isolated in Trinidad and Tobago in 1955, OROV is one of the most commonly identified arboviruses in Brazil and has expanded progressively across the Amazon basin and multiple regions in Central and South America. Autochthonous cases in 2025 have been reported in Brazil, Colombia, Cuba, Guyana, Peru, Panama and Venezuela [1,2,3]. Concern has been raised throughout other regions due to the presence of imported cases and the risk of geographical expansion [4,5].

It is a single-stranded RNA virus composed of three genomic segments—L, M, and S—within a helical nucleocapsid that enables viral protein synthesis [6]. Its segmented genome allows reassortment, raising concerns about potential shifts in pathogenicity or virulence, as seen in related viruses like Ngari, which caused fatal hemorrhagic fever [7,8]. Reassortant variants form when a host is coinfected by two related viruses [7]. To date, three reassortant OROV variants have been identified: Madre de Dios, Iquitos, and Perdões [9]. However, none of these variants have produced significant changes in the virus’s clinical or epidemiological behavior.

OROV is maintained through dual transmission cycles involving multiple arthropod vectors and vertebrate hosts such as sloths and different species of non-human primates and birds. Its main urban vector is the midge *Culicoides paraensis* due to its abundance in densely populated, water-rich environments, but additional competent vectors—such as *Aedes serratus*, *Coquillettidia venezuelensis*, *Culex quinquefasciatus* and several *Culicoides* species—support transmission in sylvatic ecosystems [10]. Although person-to-person spread has not been documented, vertical transmission occurs, and the presence of infectious virus in semen raises theoretical concerns about sexual transmission [11]. However, no sexual, transfusional, or transplant-associated cases have been confirmed to date.

Clinically, outbreaks of Oropouche fever exhibit seasonal behavior, with higher incidence during the rainy season. Following exposure, an incubation period of approximately 4–8 days precedes the onset of acute symptoms, which commonly resemble those of other arboviral infections: fever, headache, myalgia, arthralgia, anorexia, photophobia, nausea, vomiting, and chills. The published cohorts from the most recent outbreaks show general symptoms, with fever, headache, and myalgia being the most common, although the findings are not geographically homogeneous, as reports from other regions (Manaus, Brazil) show a higher incidence of rash, retro-orbital pain, and arthralgia than the Peruvian cohorts [12,13,14]. Concern has been raised as reports of cases with neurological involvement (mainly meningitis, encephalitis and Guillain-Barré Syndrome) and cases involving maternal-fetal involvement have been published [15,16,17]. Although mortality remains low, severe and rapidly progressive hemorrhagic cases resembling severe dengue have been reported in previously healthy individuals [18].

In Peru, OROV was first detected in the 1990s, and the Amazonian region of Loreto is currently the most affected area [19]. Since then, sporadic cases and outbreaks have been detected until, in 2016, the largest outbreak reported up to that time was notified in the regions of Cusco and Madre de Dios, with 57 and 120 cases respectively [20]. Subsequently, incidence decreased, with reports of sporadic cases, until the end of 2023 and the beginning of 2024, when the largest outbreak ever recorded in Peru was once again documented, coinciding with the outbreak that spread throughout South America caused by novel reassortant strains of the virus [21]. Since that outbreak, the current OROV-specific epidemiological surveillance system was implemented in Peru. In that year, there were 1263 confirmed cases nationwide, with more than 50% reported from the Loreto department, exceeding expected population-adjusted incidence compared with previous years [3,14]. Contributing factors include climate variability, flooding patterns, changes in human mobility, and ecological conditions favorable to *Culicoides paraensis*, the main urban vector capable of transmitting OROV from humans during viremia [10]. A case series from December 2023 through September 2024 in Loreto has been published recently highlighting OROV predominance in the Loreto region and including a notable pediatric cohort. The study exhibited a hospitalization rate of 2%, lower than the one in Brazilian reports, suggesting a milder disease course potentially related to regional viral strains or host factors [14].

In the present study we aimed to describe the clinical and epidemiological profile of confirmed Oropouche fever cases from October 2024 to March 2025 in the Loreto region of Peru. We specifically aimed to characterize demographic and clinical features, symptom patterns, and disease severity, with a special focus on potential atypical manifestations, to identify factors associated with hospitalization and to examine clinical outcomes among pediatric patients and pregnant women.

## 2. Materials and Methods

### 2.1. Study Design and Setting

Observational retrospective study conducted in the Loreto Region, a department of the Republic of Peru, located in the northern part of the country and sharing borders with Ecuador, Colombia and Brazil. It is the country’s largest department by area and one of the least densely populated, with a total population of 1.06 million inhabitants in 2023 [22]. Its territory is largely composed of a vast jungle lowland, with an average altitude of 50–100 m above sea level, where the Amazon River and its numerous tributaries originate and flow. The region is characterized by a humid tropical climate with an average annual temperature of 27 °C. The rainy season spans the months of December to May, with the rest of the year constituting the dry season. Because most of the territory is covered by dense rainforest with difficult access to resources and remote communications, more than half of the population is concentrated in urban centers, with Iquitos (Maynas province) being the most populated city, with nearly 500,000 inhabitants, and Nauta (in the Loreto province, sharing the name with the Department) the second most-populated with 34,000 inhabitants. The rest of the population is spread across the vast territory in small rural settlements with difficult access to resources and poor communications with major cities. Iquitos is a city bordered by the Amazon River and its tributaries, the Itaya and Nanay rivers, which experiences periodic flooding due to changes in their water levels during the rainy season, creating an environment highly conducive to the development of vectors and the transmission of infectious diseases.

### 2.2. Case Definitions [23]

- Suspected Oropouche Case: a person presenting with acute onset fever (or history of fever) of up to 5 days of evolution associated with severe headache and two or more of the following manifestations: (i) myalgia or arthralgia (ii) chills (iii) photophobia (iv) dizziness (v) retro-orbital pain (vi) nausea, vomiting, or diarrhea, (vii) any central nervous system (CNS) manifestation (diplopia, paresthesia, meningitis, encephalitis, meningoencephalitis).
- Probable Oropouche Case: any suspected case that also meets at least one of the following criteria: (i) lives in or has traveled to an area with confirmed transmission of Oropouche cases, (ii) has an epidemiological link to a confirmed Oropouche case, (iii) has a positive Oropouche ELISA IgM test result.
- Confirmed Oropouche Case: any suspected case with a positive result for Oropouche virus (OROV) detection (viral RNA via RT-qPCR or viral antigens) or presenting with antibody seroconversion or a fourfold or greater increase in antibody titer in paired samples taken more than 7–10 days apart (with a convalescent sample taken more than 14 days after symptom onset).
- Severe Oropouche: a case presenting with any of the described signs of severity (weak pulse, cold extremities, pulse pressure < 20 mmHg, shock, severe bleeding, Glasgow Coma Scale < 15) or leading to the patient’s death.

### 2.3. Data Collection and Variables

Information was retrospectively collected from the “Clinical-Epidemiological Investigation Forms for the Surveillance of Dengue, Chikungunya, Zika, Yellow Fever, and other Arboviruses” provided by the Ministry of Health of Peru. These forms correspond to suspected arboviral disease cases that initially test negative on routine assays for other arboviruses (DENV, Zika, etc.), and are sent to Lima for RT-qPCR analysis for pathogen detection for epidemiological surveillance. Authorization was requested from the Epidemiological Surveillance Unit of the Regional Health Directorate of Loreto (Dirección Regional de Salud de Loreto) to access the aforementioned forms. The information was recorded in an anonymized database created for this purpose using IBM SPSS Statistics software version 25.0 (IBM, Madrid, Spain). Obstetrical data for pregnant patients included in the study were collected from their clinical records and through home visits.

Variables analyzed included age, sex, geographic location, clinical symptoms, alarm signs (severe abdominal pain, dyspnea, effusions, persistent vomiting, hypothermia, oliguria, hepatomegaly, jaundice, increased hematocrit and altered mental status), severity markers (weak pulse, cyanosis, differential blood pressure < 20 mmHg, organ failure, severe bleeding), hospitalization, pregnancy status, and coinfections. Due to limited data availability, laboratory parameters were not analyzed.

### 2.4. Study Subjects

Inclusion Criteria: every adult or child that is a confirmed Oropouche case diagnosed by RT-qPCR between October 2024 and March 2025 in the Loreto Region, Peru.

Exclusion Criteria: probable or suspected OROV cases, cases negative for OROV or incomplete essential data available.

The study sample size was not predetermined. All cases meeting the inclusion criteria during the established period were included.

### 2.5. Statistical Analysis Strategy

Frequency variables were expressed as absolute numbers, percentages, and confidence intervals. Continuous variables were expressed as mean (standard deviation) and median (interquartile range), depending on the result of the appropriate normality test (Kolmogorov–Smirnov or Shapiro–Wilk test).

Comparisons were performed between pediatric (<14 years) and adult groups, between males and females, and between hospitalized and non-hospitalized patients.

For comparisons, the Chi-squared test and Fisher’s exact test were employed for qualitative variables, while quantitative ones were analyzed using Student’s *t*-test or the Mann–Whitney U test, depending on whether they follow a parametric or non-parametric distribution, respectively Missing data were not imputed and are left as lost values, with the number of valid observations used for the analysis indicated for each variable. The level of statistical significance is stated at *p* < 0.05. Data were analyzed using the statistical analysis software IBM SPSS Statistics version 25.0 (IBM, Madrid, Spain). The choropleth map was created using the Datawrapper software tool (Datawrapper GmbH, Berlin, Germany) (web: https://www.datawrapper.de/).

### 2.6. Ethical Considerations

The study investigators have adhered to the current revision of the Declaration of Helsinki (2024) and the data protection regulations in both Peru and Spain. The study was evaluated and approved in May 2025 by the Institutional Ethics and Research Committee of the Loreto Regional Hospital (ID-041-CIEI-2025) and holds certification from the Office of Responsible Research at the Miguel Hernández University. Data were pseudo-anonymized. Waiver of informed consent was requested and obtained.

## 3. Results

A total of 100 laboratory confirmed cases of Oropouche fever were identified. Fifty-nine patients (59%) were female and forty-one were male (41%), and the median age was 28 years (interquartile range 14–40). Twenty-five cases occurred in pediatric patients (<14 years old). Six cases involved pregnant women. All cases were locally acquired, no imported cases were reported.

### 3.1. Temporal and Geographic Distribution

**Figure 1:**
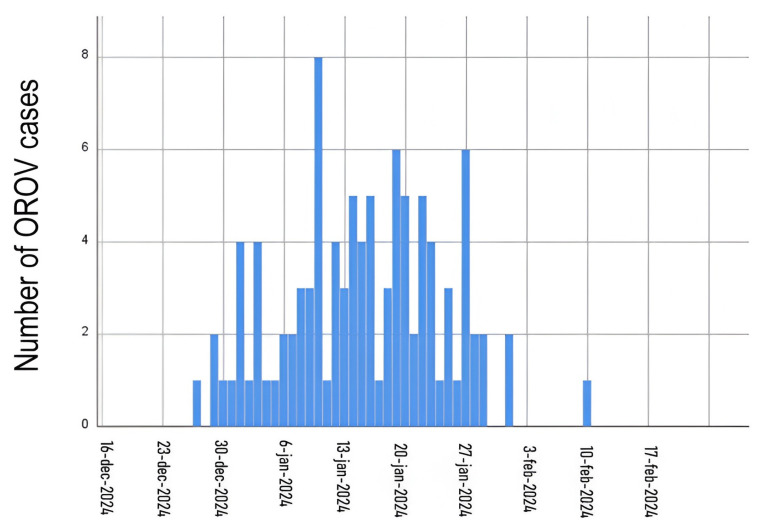
Temporal distribution of confirmed cases of Oropouche fever in Departamento de Loreto, Peru, from October 2024 to March 2025.

The earliest symptom onset was recorded on 29 December 2024 and the last on 10 February 2025, demonstrating concentrated transmission over slightly more than five weeks (Figure 1). The incidence peak occurred on 10 January.

**Figure 2:**
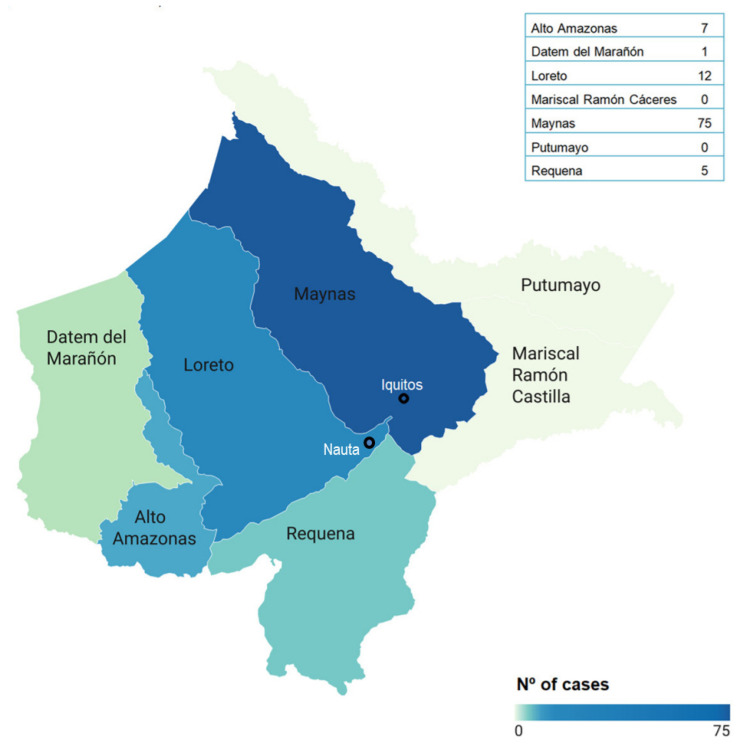
Geographical distribution of confirmed cases of Oropouche fever in Departamento de Loreto, Peru, from October 2024 to March 2025.

Most cases originated from the province of Maynas (75%) where Iquitos (Department’s most populated city) is located, followed by Loreto (12%) (where is the second biggest city of the Department, Nauta) and Alto Amazonas (7%), reflecting both population distribution and access to testing services (Figure 2).

### 3.2. Diagnostic Confirmation and Coinfections

All cases were confirmed through real-time polymerase chain reaction (RT-qPCR) performed on serum samples, with 6 cases having also a positive Oropouche IgM. Seventy patients hold registration of DENV tests among our data through ELISA NS1, ELISA IgM, RT-qPCR or rapid-tests, among whom one patient presented confirmed dengue coinfection (RT-qPCR positive), two presented probable coinfection (2 ELISA NS1 positive) and one had ELISA IgM positive, not being able to confirm coinfection due to lack of seroconversion record and the possibility of cross-reactivity. No chikungunya or Zika virus coinfections were detected.

### 3.3. Oropouche Fever Clinical Presentation

The most frequently reported symptom was fever (88%), followed by headache (78%), myalgia (72%), arthralgia (65%), nausea or vomiting (42%), back pain (38%), and retro-ocular pain (28%). Rash was reported only in 2% of cases, and conjunctivitis was absent. Alarm signs were present in 10 patients, consisting on mainly digestive symptoms, with nausea and vomiting affecting 7 patients and abdominal pain in all of them. No patient reported neurological symptoms, hemorrhagic manifestations, severe dehydration, tachypnea, hemodynamic instability, or laboratory-indicated liver failure (Table 1).

### 3.4. Oropouche Fever Hospitalizations

Eight patients (8%) required hospitalization. Factors significantly associated with hospitalization included persistent vomiting (OR 75.0; 95%CI: 10.1–555.8, *p* < 0.0001), pregnancy (OR 17.8; 95%CI: 2.8–111.7, *p* = 0.006), intense abdominal pain (OR 66.0; 95%CI: 9.9–436.0, *p* < 0.0001) and arthralgia (*p* = 0.027). All hospitalized patients were adults, mainly female. No patient developed severe disease or required intensive care (Table 2).

### 3.5. Age and Gender Subgroup Analysis

The incidence of arthralgia was more frequent among women (OR 2.8; 1.14–6.89, *p* = 0.023) with no other significant differences in symptoms between males and females (Appendix A, Table A1). Children significantly suffered less from abdominal pain (OR 0.87; 0.79–0.95, *p* = 0.048) (Appendix A, Table A2).

### 3.6. Pregnancy Outcomes

Six confirmed cases occurred in pregnant women. Three experienced complications: one case of premature rupture of membranes, followed by neonatal jaundice requiring phototherapy, one case of preeclampsia requiring caesarean delivery and one case of fetal macrosomia (Table 3). There were no cases of miscarriage, congenital malformations, congenital infection, or neonatal mortality. Complications were reported in patients infected in the first and second trimester, while those occurring in the third trimester did not lead to adverse outcomes. All newborns were discharged in stable condition.

### 3.7. Atypical and Neurological Presentations

No atypical or neurological manifestations were identified. No cases of meningitis, encephalitis, Guillain–Barre syndrome, seizures, or hemorrhagic disease were reported.

## 4. Discussion

Our study summarizes findings from the Oropouche fever epidemic in the Department of Loreto, Peru, based on an analysis of 100 RT-qPCR-confirmed cases between October 2024 and March 2025. Most cases occurred in adults, and no severe Oropouche fever presentations or deaths were identified.

OROV is an increasingly relevant arbovirus in the differential diagnosis of acute febrile illness in the Americas. Epidemics have expanded throughout Central and South America in recent years, although surveillance systems in many countries remain suboptimal, likely underestimating the true burden of disease [24,25]. In Loreto, 496 confirmed cases were reported between December 2023 and September 2024, mainly (72.9%) in the Maynas province. The early 2025 wave shows fewer cases, concentrated almost entirely in January, consistent with the data published in the last Epidemiological Update on Oropouche in the Americas Region by the PAHO showing a concentration of cases in the country within the first 11 epidemiological weeks [3]. The geographic distribution is consistent with previous years. The low numbers reported from remote districts may suggest improved adaptation of vectors to urban areas, although this pattern could also be influenced by underreporting related to barriers in access to health services and limited molecular diagnostic capacity.

In Loreto, ten different strains of the virus have been identified between 2023 and 2024. The reported circulating strains from this region are distinct from those causing disease in the neighbor country Brazil despite the geographical proximity, and more closely related to those causing outbreaks in Colombia in 2020 and Ecuador in 2016 [26]. Unfortunately, no clinical cohorts of patients from these outbreaks have been published to date that would allow us to perform a comparative analysis. In our study we could not access the information regarding the strain type of the analyzed samples.

Diagnostic capacity for OROV in Loreto is limited. IgM ELISA availability is scarce and irregular, and most diagnoses rely on RT-qPCR, which is-performed only in those cases with an initial negative test for dengue- at a reference laboratory in Lima. This testing algorithm likely contributes to underdiagnosis, particularly of coinfections with DENV. Cheaper, point-of-care diagnostic tests are much needed and could improve both clinical and public health outcomes.

The clinical profile in our cohort aligns with existing literature, with fever, headache, and myalgia being the predominant symptoms [16,27]. Rash was uncommon (2%, none of them coinfected), as was conjunctivitis, suggesting that their presence may point to alternative arboviral etiologies such as DENV or Zika [28], though no clinical pattern reliably differentiates Oropouche fever from other arboviruses without laboratory confirmation [12,29]. A previous study conducted in the region reported a similar clinical presentation, with fever, myalgia, and headache as the predominant symptoms. However, that study described a higher incidence of rash (4.0%) and conjunctivitis (1.8%), along with a lower frequency of warning signs—persistent abdominal pain (1.6% vs. 10.0%) and persistent vomiting (1.2% vs. 7.0%)—and a lower proportion of hospitalized patients (2.0% vs. 8.0%). In contrast, the outbreak described in our study appears to show a trend toward greater relative severity [14]. Oropouche fever symptoms generally last 2–4 days; however, approximately 60% of patients experience a biphasic course, with recurrence of symptoms 7–10 days after the initial febrile episode [7]. Sadly, the clinical–epidemiological forms do not record information on this biphasic pattern.

Severe neurological involvement has also been documented, including encephalitis, meningoencephalitis, and post-infectious Guillain–Barré syndrome [30,31]. None of these complications were recorded in our study. Neurological complications in OROV infection are rarely reported in cohorts and are mostly described as isolated case reports. Consequently, the true incidence of meningitis, encephalitis, and Guillain–Barré syndrome (GBS) remains unknown but appears to be low, consistent with the absence of such cases in our cohort. Mild presentations of meningitis or encephalitis may go unnoticed in low-resource settings, while GBS—clinically indistinguishable from other causes and only recently linked to OROV—may not have been routinely considered, contributing to potential underdiagnosis. In Loreto, hospitalized cases would likely have been captured through mandatory reporting, although under-recognition among individuals who did not seek care cannot be excluded.

No patient in the study exhibited established severity criteria. The only warning signs observed were severe abdominal pain and persistent vomiting—criteria extrapolated from the non-related DENV classifications because of the paucity of reported severe Oropouche cases. Consequently, the applicability of these warning signs to Oropouche fever remains uncertain and will require reassessment as further evidence accumulates, with the aim of defining OROV-specific risk factors and severity criteria as clinical experience and research on this disease expand. In the 2024 cohort hospitalization was also primarily driven by severe arthralgia, persistent abdominal pain, and vomiting [14]. To date, no fatal cases of Oropouche fever have been reported in the Loreto region; however, in 2024, Brazil reported the first severe Oropouche cases with hemorrhagic manifestations and fatal outcomes in two young patients without prior comorbidities, who experienced a rapidly progressive clinical course [18]. These newly detected cases heighten the alert surrounding this disease and should prompt reinforcement of epidemiological surveillance systems, especially given the virus’s high reassortment potential [7].

Recent concern has focused on maternal–fetal transmission, following reports of spontaneous abortion, congenital malformations such as microcephaly, and fetal death [32,33,34]. In our study, six cases occurred during pregnancy; three were associated with obstetric complications, all in the first and second trimester (macrosomia, premature rupture of membranes and preeclampsia requiring caesarean delivery). No fetal malformations or adverse neonatal outcomes were documented. Complications were absent among infections occurring in the third trimester, consistent with observations of increased vulnerability earlier in gestation [35,36].

The study has several inherent limitations. Its retrospective design and reliance on routine surveillance forms resulted in the incomplete recording of several key variables. As mentioned above, many of the variables collected are extrapolated from the DENV alarm criteria, so we were unable to record other variables that might be of greater interest in the specific case of Oropouche fever. This fact may have influenced the interpretation of the data. Future studies, ideally prospective and with a larger sample size, could help to establish disease-specific signs, symptoms or alarm factors that reduce the resulting information biases. Unfortunately, we did not have access to data on the number of probable or suspected Oropouche cases or on the total number of patients tested. Consequently, we are unable to assess if certain demographic groups were over-represented in testing or confirmed cases. In addition, due to the cross-sectional nature of the epidemiological investigation forms and the difficulty in detecting and following up cases because of the region’s idiosyncrasies, no confirmed Oropouche cases based on seroconversion could be obtained. Furthermore, restricted diagnostic access in the region may have introduced a sampling bias, potentially skewing the distribution of reported cases toward urban centers where testing is more readily available. The overall small sample size, particularly among the specific subgroup of pregnant women, restricts the generalizability of the findings and its statistical power with wide confidence intervals for the calculated odds ratio may have failed to detect some associations. Finally, the lack of comprehensive laboratory and viral sequencing data significantly limited the ability to thoroughly analyze the determinants of disease severity and explore the potential contribution of Oropouche virus strain variation to the observed clinical outcomes. However, it complements information of previous studies in the region of our group [14] and adds follow-up information on pregnancies.

Despite its limitations, our study provides a detailed clinical and epidemiological description of the recent 2025 outbreak in Loreto, describing a cohort of patients with confirmed OROV diagnosis and including specific comparisons between subgroups by sex and age, as well as identifying risk factors for hospitalization not previously reported in the literature. We provide a description of a series of cases of pregnant patients that shed new light on fetal Oropouche and its relationship with pregnancy, and finally, we report on the spatial-temporal evolution of cases in the Loreto region, with important epidemiological considerations for monitoring the evolution of the disease in the region.

## 5. Conclusions

From October 2024 to March 2025, 100 RT-qPCR confirmed OROV cases were recorded in Loreto, representing a new outbreak with similar but somehow more severe clinical presentation compared to the 2024 epidemic. No severe or fatal cases occurred. Hospitalization was associated with abdominal pain, persistent vomiting, arthralgia, and pregnancy. Six infections occurred in pregnant women, of which two led to obstetric complications but without fetal malformation or pregnancy loss. No neurological or atypical presentations were observed.

Our findings highlight the expanding epidemiological relevance of OROV, the need for enhanced diagnostic capacity, and the importance of robust surveillance systems to detect severe disease and monitor vulnerable populations such as pregnant women.

## Figures and Tables

**Table 1:** Clinical and demographical characteristics of cases of Oropouche virus disease in Loreto, Perú October 2024–March 2025.

Characteristics	N (%)
Gender	
Women	59 (59%)
Male	41 (41%)
Pregnant women	6 (6%)
Age, years old; median (IQR)	28 (14.25–40.75)
Origin: urban	81 (81%)
Documented previous dengue infection	4 (4%)
History of the yellow fever vaccine	9 (9%)
Comorbidities	3 (3%)
**Symptoms**	
Fever	88 (88%)
Arthralgia	65 (65%)
Arthralgia (hands)	48 (48%)
Arthralgia (feet)	52 (52%)
Myalgia	72 (72%)
Headache	78 (78%)
Retro-ocular pain	28 (28%)
Back pain	34 (34%)
Rash	2 (2%)
Conjunctivitis	0 (0%)
Nausea or vomiting	42 (42%)
**Alarm Signs**	
Abdominal pain	10 (10%)
Dyspnea or chest pain	0 (0%)
Effusions	0 (0%)
Persistent vomiting	7 (7%)
Hypothermia	0 (0%)
Oliguria	0 (0%)
Hepatomegaly	0 (0%)
Jaundice	0 (0%)
Neurological symptoms	0 (0%)
High hematocrit	0 (0%)
**Severity Markers**	
Oropouche severe	0 (0%)
Hospitalization	8 (8%)
Death	0 (0%)

**Table 2:** Factors associated with hospitalization among patients with Oropouche infection in the Department of Loreto, October 2024–March 2025.

Characteristics	Hospitalized n/N (%)	Not Hospitalized n/N (%)	OR (95%CI)	*p*-Value
Abdominal pain	6/8 (75%)	4/92 (4.3%)	66.0 (9.9–436.0)	**<0.0001**
Persistent vomiting	5/8 (62.5%)	2/92 (2.2%)	75.0 (10.1–555.8)	**<0.0001**
Arthralgia	8/8 (100.0%)	57/92 (62.0%)	NC	**0.027**
Fever	7/8 (87.5%)	81/92 (88.0%)	0.95 (0.11–8.47)	0.655
Myalgia	8/8 (100.0%)	64/92 (69.6%)	NC	0.064
Headache	7/8 (87.5%)	71/92 (77.2%)	2.07 (0.24–17.79)	0.438
Back pain	5/8 (62.5%)	29/92 (31.5%)	3.62 (0.81–16.18)	0.086
Retro-ocular pain	2/8 (25.0%)	26/92 (28.3%)	0.85 (0.16–4.46)	0.603
Rash	0/8 (0.0%)	2/92 (2.2%)	NC	0.846
Conjunctivitis	0/8 (0.0%)	0/92 (0.0%)	NC	NC
Nausea/vomiting	5/8 (62.5%)	37/92 (40.2%)	2.47 (0.56–11.00)	0.197
Gender (woman)	7/8 (87.5%)	52/92 (56.5%)	3.48 (0.55–22.08)	0.087
Origin (urban)	5/8 (62.5%)	76/92 (82.6%)	2.85 (0.62–13.16)	0.174
Age (adults)	8/8 (100.0%)	67/92 (72.8%)	NC	0.091
Pregnancy	3/8 (37.5%)	3/92 (3.3%)	17.8 (2.8–111.7)	**0.006**

NC: not calculable. **In bold**: statistically significant. OR: odds ratio. CI: confidence interval.

**Table 3:** Description of Oropouche cases in pregnant patients in the Department of Loreto from October 2024 to March 2025.

Case Number	Age	Weeks of Pregnancy at Diagnosis	Delivery Week	Newborn Weight	Complications	Description
Case 1	33	20	35 + 2	2705 g	Yes	Premature rupture of membranes, neonatal hyperbilirubinemia
Case 2	19	34	39	3200 g	No	
Case 3	22	37	40	3700 g	No	
Case 4	15	35	40	3900 g	No	
Case 5	25	26	37	2700 g	Yes	Preeclampsia, caesarean section
Case 6	16	5	37	4500 g	Yes	Macrosomia

## Data Availability

The original data presented in the study are openly available in Zenodo at DOI: 10.5281/zenodo.17801182.

## Abbreviations

The following abbreviations are used in this manuscript:

OROV	Oropouche virus
DENV	Dengue virus
RT-qPCR	real-time polymerase chain reaction
PAHO	Pan American Health Organization

## Appendix A

**Table A1:** Differences in clinical presentation between men and female among Oropouche cases in the Department of Loreto, October 2024–March 2025.

Characteristics	Women n/N (%)	Men n/N (%)	OR (95%CI)	*p*-Value
Arthralgia	32/41 (78.0%)	33/59 (55.9%)	2.80 (1.13–6.89)	**0.018**
Fever	33/41 (80.5%)	55/59 (93.2%)	0.30 (0.08–1.07)	0.054
Nausea and vomiting	14/41 (34.1%)	28/59 (47.5%)	0.57 (0.25–1.31)	0.131
Rash	0/41 (0.0%)	2/59 (3.4%)	NC	0.346
Back pain	10/41 (24.4%)	24/59 (40.7%)	0.47 (0.19–1.14)	0.069
Retro-ocular pain	8/41 (19.5%)	20/59 (33.9%)	0.47 (0.18–1.21)	0.087
Headache	32/41 (78.0%)	46/59 (78.0%)	1.00 (0.38–2.63)	0.596
Myalgia	29/41 (70.7%)	43/59 (72.9%)	0.89 (0.37–2.17)	0.494
Abdominal pain	2/41 (4.9%)	8/59 (13.6%)	0.33 (0.07–1.63)	0.138
Persistent vomiting	1/41 (2.4%)	6/59 (10.2%)	0.22 (0.02–1.91)	0.137
Hospitalized	1/41 (2.4%)	7/59 (11.9%)	0.19 (0.02–1.57)	0.087

NC: not calculable. **In bold**: statistically significant.

**Table A2:** Differences in clinical presentation between children and adults among Oropouche cases in the Department of Loreto, October 2024–March 2025.

Characteristics	Children (≤14 Years Old) n/N (%)	Adults n/N (%)	OR (95%CI)	*p*-Value
Arthralgia	13/25 (52.0%)	52/75 (69.3%)	0.47 (0.19–1.21)	0.093
Fever	21/25 (84.0%)	67/75 (89.3%)	0.63 (0.17–2.3)	0.347
Nausea and vomiting	14/25 (56.0%)	28/75 (37.3%)	2.13 (0.85–5.35)	0.081
Rash	0/25 (0.0%)	2/75 (2.7%)	NC	0.561
Back pain	8/25 (32.0%)	26/75 (34.7%)	0.88 (0.34–2.33)	0.505
Retro-ocular pain	4/25 (16.0%)	24/75 (32.0%)	0.41 (0.13–1.31)	0.096
Headache	20/25 (80.0%)	58/75 (77.3%)	1.17 (0.38–3.59)	0.511
Myalgia	18/25 (75.0%)	54/75 (72.0%)	1.00 (0.36–2.74)	0.594
Abdominal pain	0/25 (0.0%)	10/75 (13.3%)	0.87 (0.79–0.95)	**0.048**
Persistent vomiting	0/25 (0.0%)	7/75 (9.3%)	0.91 (0.84–0.97)	0.124
Hospitalized	0/25 (0.0%)	8/75 (10.7%)	NC	0.091

NC: not calculable. **In bold**: statistically significant.
